# Supplementary material for: Status and subjective well-being: A conceptual replication and extension of Anderson et al. (2012)
Source: PLoS One. 2024 Sep 18;19(9):e0309135. doi: 10.1371/journal.pone.0309135 (PMC11410214; doi:10.1371/journal.pone.0309135)
Supplement: S1 Appendix — (DOCX) [file pone.0309135.s001.docx]

# **Appendix A. Feedback and Rating Task**

[All participants were presented with the following information]

**Feedback and Ratings Task – Ratings**

Thank you for completing Questionnaire 1. The other members of your group also completed the same Questionnaire. Summaries of each of their personality scores and emotion-recognition skills are shown in the table below. (Note, your scores are not shown below, but will be shown to your fellow group members).

*Agreableness* reflects the person’s kindness, generosity, and tender-minded concern for others.

*Conscientiousness* reflects the person’s work ethic, dutifulness, and attention to detail.

*Extraversion* reflects the person’s sociability, talkativeness, and assertiveness.

*Emotion-recognition skill* reflects the person’s ability to read others’ emotions from facial expressions.

|  | Agreeableness | Conscientiousness | Extraversion | Emotion-Recognition Skill |
| --- | --- | --- | --- | --- |
| Possible range: | 1 (low) – 7 (high) | 1 (low) – 7 (high) | 1 (low) – 7 (high) | 0 (low) – 24 (high) |
| Member A | 3.5 | 6.0 | 4.0 | 17 |
| Member B (you) | - | - | - | - |
| Member C | 4.0 | 4.5 | 5.5 | 9 |
| Member D | 1.5 | 3.0 | 5.5 | 4 |
| Member E | 6.0 | 3.0 | 2.5 | 20 |

**Please review the scores above carefully. Based on these scores, rate the status of each member of the group using the scale below.** That is, how much should each member be respected and admired in the group, how much should they lead the group’s task, and how much should others voluntarily defer to them?

Keep in mind the group will complete a series of decision-making tasks and solve challenging problems together. (Note, you will not be asked to rate your own status.)

The overall status ratings of each group member will be used to determine which person will be selected as the leader on a group exercise you will complete later in this study.

**How much status should this person receive?**

|  | None  1 | 2 | 3 | A moderate amount  4 | 5 | 6 | A lot  7 |
| --- | --- | --- | --- | --- | --- | --- | --- |
| Member A | ο | ο | ο | ο | ο | ο | ο |
| Member B | ο | ο | ο | ο | ο | ο | ο |
| Member C | ο | ο | ο | ο | ο | ο | ο |
| Member D | ο | ο | ο | ο | ο | ο | ο |

Please wait while the other participants complete their ratings.

Once everyone has completed their ratings and the results have been processed, the experimenter will give you a code to write in the box below.

# **Appendix B. Status Manipulation**

[All participants were presented with the following introduction:]

**Feedback and Ratings Task - your feedback**

Thank you for rating the other members of your group. Each other member of your group also completed the same ratings you just did. Below is a summary of the ratings you received from them as well as the group’s overall average. Later in the study you will be told which group member has been selected as the leader based on these ratings.

[Only participants in Condition 1 (Own status low, Other status low) saw the following:]

| **Range of possible status ratings**  (i.e. the minimum and maximum possible status ratings that a person could receive) | Minimum: 1  Maximum: 7 |
| --- | --- |
| **Your status**  (i.e. the median status rating you were given by other members of your group) | **4** |
| **Other group members’ average status**  (i.e. the median status ratings that all the other members of your group received) | **4** |

The rest of your group members ranked your status as a 4, while the average ranking of your group as a whole was a 4.

Your status rating is equal to the average of your group.

Please review the information above carefully before continuing.

[Only participants in Condition 2 (Own status low, Other status high) saw the following:]

| **Range of possible status ratings**  (i.e. the minimum and maximum possible status ratings that a person could receive) | Minimum: 1  Maximum: 7 |
| --- | --- |
| **Your status**  (i.e. the median status rating you were given by other members of your group) | **4** |
| **Other group members’ average status**  (i.e. the median status ratings that all the other members of your group received) | **6** |

The rest of your group members ranked your status as a 4, while the average ranking of your group as a whole was a 6.

Your status rating is below to the average of your group.

Please review the information above carefully before continuing.

[Only participants in Condition 3 (Own status high, Other status low) saw the following:]

| **Range of possible status ratings**  (i.e. the minimum and maximum possible status ratings that a person could receive) | Minimum: 1  Maximum: 7 |
| --- | --- |
| **Your status**  (i.e. the median status rating you were given by other members of your group) | **6** |
| **Other group members’ average status**  (i.e. the median status ratings that all the other members of your group received) | **4** |

The rest of your group members ranked your status as a 6, while the average ranking of your group as a whole was a 4.

Your status rating is above the average of your group.

Please review the information above carefully before continuing.

[Only participants in Condition 4 (Own status high, Other status high) saw the following:]

| **Range of possible status ratings**  (i.e. the minimum and maximum possible status ratings that a person could receive) | Minimum: 1  Maximum: 7 |
| --- | --- |
| **Your status**  (i.e. the median status rating you were given by other members of your group) | **6** |
| **Other group members’ average status**  (i.e. the median status ratings that all the other members of your group received) | **6** |

The rest of your group members ranked your status as a 6, while the average ranking of your group as a whole was a 6.

Your status rating is equal to the average of your group.

Please review the information above carefully before continuing.
