# Supplementary material for: Status and subjective well-being: A conceptual replication and extension of Anderson et al. (2012)
Source: PLoS One. 2024 Sep 18;19(9):e0309135. doi: 10.1371/journal.pone.0309135 (PMC11410214; doi:10.1371/journal.pone.0309135)
Supplement: Attachment — Submitted filename: ResponseToReviewers.2024.05.10.docx [file pone.0309135.s002.docx]

**Response to Reviewers**

[Original text shown indented in italics]

*PONE-D-24-10935*

*Status and subjective well-being: A conceptual replication and extension of Anderson et al. (2012)*

*PLOS ONE*

*Dear* [ANONYMIZED]*,*

*Thank you for submitting your manuscript to PLOS ONE. After careful consideration, we feel that it has merit but does not fully meet PLOS ONE’s publication criteria as it currently stands. Therefore, we invite you to submit a revised version of the manuscript that addresses the points raised during the review process.*

*The revised version should address all comments. You may also note that unemployment is an important determinant of subjective well-being (https://doi.org/10.1002/hec.1361).*

*Please submit your revised manuscript by Jun 14 2024 11:59PM. If you will need more time than this to complete your revisions, please reply to this message or contact the journal office at* [*plosone@plos.org*](mailto:plosone@plos.org)*. When you're ready to submit your revision, log on to https://www.editorialmanager.com/pone/ and select the 'Submissions Needing Revision' folder to locate your manuscript file.*

*Please include the following items when submitting your revised manuscript:*

- *A rebuttal letter that responds to each point raised by the academic editor and reviewer(s). You should upload this letter as a separate file labeled 'Response to Reviewers'.*
- *A marked-up copy of your manuscript that highlights changes made to the original version. You should upload this as a separate file labeled 'Revised Manuscript with Track Changes'.*
- *An unmarked version of your revised paper without tracked changes. You should upload this as a separate file labeled 'Manuscript'.*

*If you would like to make changes to your financial disclosure, please include your updated statement in your cover letter. Guidelines for resubmitting your figure files are available below the reviewer comments at the end of this letter.*

*If applicable, we recommend that you deposit your laboratory protocols in protocols.io to enhance the reproducibility of your results. Protocols.io assigns your protocol its own identifier (DOI) so that it can be cited independently in the future. For instructions see:* [*https://journals.plos.org/plosone/s/submission-guidelines#loc-laboratory-protocols*](https://nam12.safelinks.protection.outlook.com/?url=https%3A%2F%2Fjournals.plos.org%2Fplosone%2Fs%2Fsubmission-guidelines%23loc-laboratory-protocols&data=05%7C02%7Cjdh362%40cornell.edu%7C16a98a46a4b54c331ba108dc69e97692%7C5d7e43661b9b45cf8e79b14b27df46e1%7C0%7C0%7C638501699849678666%7CUnknown%7CTWFpbGZsb3d8eyJWIjoiMC4wLjAwMDAiLCJQIjoiV2luMzIiLCJBTiI6Ik1haWwiLCJXVCI6Mn0%3D%7C0%7C%7C%7C&sdata=02JqA649L4NTvBJ%2Br7WxsUWdxjMRS1kPMATx2mR8JBM%3D&reserved=0)*. Additionally, PLOS ONE offers an option for publishing peer-reviewed Lab Protocol articles, which describe protocols hosted on protocols.io. Read more information on sharing protocols at* [*https://plos.org/protocols?utm_medium=editorial-email&utm_source=authorletters&utm_campaign=protocols*](https://nam12.safelinks.protection.outlook.com/?url=https%3A%2F%2Fplos.org%2Fprotocols%3Futm_medium%3Deditorial-email%26utm_source%3Dauthorletters%26utm_campaign%3Dprotocols&data=05%7C02%7Cjdh362%40cornell.edu%7C16a98a46a4b54c331ba108dc69e97692%7C5d7e43661b9b45cf8e79b14b27df46e1%7C0%7C0%7C638501699849688034%7CUnknown%7CTWFpbGZsb3d8eyJWIjoiMC4wLjAwMDAiLCJQIjoiV2luMzIiLCJBTiI6Ik1haWwiLCJXVCI6Mn0%3D%7C0%7C%7C%7C&sdata=jkSn9Xrmhi0slSGy2hXNx0Q%2FAF%2BtiPYzHZKzf1hDW%2B4%3D&reserved=0)*.*

*We look forward to receiving your revised manuscript.*

*Kind regards,*

*Petri Böckerman*

*Academic Editor*

*PLOS ONE*

*Journal Requirements:*

*When submitting your revision, we need you to address these additional requirements.*

*1. Please ensure that your manuscript meets PLOS ONE's style requirements, including those for file naming. The PLOS ONE style templates can be found at*

[*https://journals.plos.org/plosone/s/file?id=wjVg/PLOSOne_formatting_sample_main_body.pdf*](https://journals.plos.org/plosone/s/file?id=wjVg/PLOSOne_formatting_sample_main_body.pdf) *and*

[*https://journals.plos.org/plosone/s/file?id=ba62/PLOSOne_formatting_sample_title_authors_affiliations.pdf*](https://journals.plos.org/plosone/s/file?id=ba62/PLOSOne_formatting_sample_title_authors_affiliations.pdf)

*2. Please include your full ethics statement in the ‘Methods’ section of your manuscript file. In your statement, please include the full name of the IRB or ethics committee who approved or waived your study, as well as whether or not you obtained informed written or verbal consent. If consent was waived for your study, please include this information in your statement as well.*

**RESPONSE:** Thank you for your and the review team’s comments and advice and for the opportunity to submit a revised manuscript. As requested we have included this Response to Reviewers as well as a marker-up and unmarked version of the revised manuscript. We confirm the following:

- We don’t wish to submit an updated financial disclosure.
- We have resubmitted our figure files in tif format per the guidelines.
- The lab protocols have been submitted to OSF (where the data had been stored) rather than protocols.io to reduce the burden on readers having to navigate between different systems.
- We have ensured the revision meets PLOSOne’s formatting guidelines. Thank you for sharing the templates.
- We have included the full ethics statement in the Methods sections of Study 1. Specifically, we have edited the prior statement to now read: “*The research in both studies was conducted in accordance with the principles expressed in the Declaration of Helsinki and the protocol was approved by The Committee for Protection of Human Subjects comprising the Institutional Review Boards of the University of California, Berkeley (IRB Approval ID: 2015-01-7044). All subjects gave their informed consent for inclusion before they participated in the study. and were then debriefed at the end of the study*.”
- We also included the citation relating to the relationship between unemployment and subjective health-assessments in the revised manuscript (Bockerman & Ilmakunnas, 2009).

**Additional Editor Comments:**

*The revised version should address all comments.*

*Comments from PLOS Editorial Office:*

*We note that one or more reviewers has recommended that you cite specific previously published works. As always, we recommend that you please review and evaluate the requested works to determine whether they are relevant and should be cited. It is not a requirement to cite these works. We appreciate your attention to this request.*

*[Note: HTML markup is below. Please do not edit.]*

**Reviewers' comments:** Thank you for clarifying.

*Reviewer's Responses to Questions*

***Comments to the Author***

*1. Is the manuscript technically sound, and do the data support the conclusions?*

*The manuscript must describe a technically sound piece of scientific research with data that supports the conclusions. Experiments must have been conducted rigorously, with appropriate controls, replication, and sample sizes. The conclusions must be drawn appropriately based on the data presented.*

*Reviewer #1: Partly*

*Reviewer #2: Yes*

*2. Has the statistical analysis been performed appropriately and rigorously?*

*Reviewer #1: Yes*

*Reviewer #2: Yes*

*3. Have the authors made all data underlying the findings in their manuscript fully available?*

*The* [*PLOS Data policy*](https://nam12.safelinks.protection.outlook.com/?url=http%3A%2F%2Fwww.plosone.org%2Fstatic%2Fpolicies.action%23sharing&data=05%7C02%7Cjdh362%40cornell.edu%7C16a98a46a4b54c331ba108dc69e97692%7C5d7e43661b9b45cf8e79b14b27df46e1%7C0%7C0%7C638501699849695624%7CUnknown%7CTWFpbGZsb3d8eyJWIjoiMC4wLjAwMDAiLCJQIjoiV2luMzIiLCJBTiI6Ik1haWwiLCJXVCI6Mn0%3D%7C0%7C%7C%7C&sdata=%2B%2FJJ1%2FfPKKJB7qHqJWWk99mwhF9iHAw4ngph74i5mMM%3D&reserved=0) *requires authors to make all data underlying the findings described in their manuscript fully available without restriction, with rare exception (please refer to the Data Availability Statement in the manuscript PDF file). The data should be provided as part of the manuscript or its supporting information, or deposited to a public repository. For example, in addition to summary statistics, the data points behind means, medians and variance measures should be available. If there are restrictions on publicly sharing data—e.g. participant privacy or use of data from a third party—those must be specified.*

*Reviewer #1: Yes*

*Reviewer #2: Yes*

*4. Is the manuscript presented in an intelligible fashion and written in standard English?*

*PLOS ONE does not copyedit accepted manuscripts, so the language in submitted articles must be clear, correct, and unambiguous. Any typographical or grammatical errors should be corrected at revision, so please note any specific errors here.*

*Reviewer #1: Yes*

*Reviewer #2: Yes*

*5. Review Comments to the Author*

*Please use the space provided to explain your answers to the questions above. You may also include additional comments for the author, including concerns about dual publication, research ethics, or publication ethics. (Please upload your review as an attachment if it exceeds 20,000 characters)*

**Reviewer 1 comments**

*Reviewer #1: The topic is fascinating, but a thorough restructuring of the content is necessary. I kindly request the esteemed author to revise the entire article with a deeper and more coherent perspective, taking into consideration the suggested changes. Afterward, please resubmit it for further review. I believe this feedback will be valuable in improving the quality of your work.*

*Best regards.*

**RESPONSE:** We have made substantial revisions to the paper, throughout all sections, based on your comments, the other reviewer’s comments, and the editor’s suggestions. We discuss below how we addressed each of your comments in turn.

*Abstract*

*1. The could benefit from a clearer structure. Consider breaking it down into distinct sections, such as Introduction, Methods, Results, and Conclusion.*

*2. Begin with a concise introduction to provide context and importance of the research question.*

*3. Provide more details about the experiments conducted, including sample size, demographics, and any relevant variables controlled for.*

*4. Expand on the implications of the findings for theory and practice.*

**RESPONSE:** Thank you for this suggestion. We examined the abstracts of several articles published in PLOS ONE and found that most of them used the same structure in the abstract that we did; fewer used the breakdown you suggested. Therefore, in keeping with what appears to be more of the norm in PLOS ONE articles, we retained the original structure of our abstract.

*introduction*

*• Consider providing a bit more context on the theoretical frameworks that underpin the relationship between status and SWB, such as social comparison theory or self-esteem theory.*

*• The introduction clearly outlines the three primary aims of the current research, including a conceptual replication of previous findings, examination of the effect of others' status on SWB, and exploration of potential interventions.*

*• To enhance the quality of your introduction, consider incorporating the following references:*

[*https://link.springer.com/article/10.1007/s10943-020-01151-z*](https://link.springer.com/article/10.1007/s10943-020-01151-z)

[*http://jpcp.uswr.ac.ir/browse.php?a_id=787&sid=1&slc_lang=fa&ftxt=0*](http://jpcp.uswr.ac.ir/browse.php?a_id=787&sid=1&slc_lang=fa&ftxt=0)

*• Conclude the introduction by summarizing the main points discussed and clearly transitioning to the subsequent sections of the paper, such as the methodology and results.*

*• Ensure the introduction sets the stage effectively for the reader and generates interest in the research questions addressed.*

**RESPONSE:** Based on your suggestions, we elaborated on why status might shape SWB, particularly the possibility that status shapes self-esteem, which in turn influences SWB. We also included a statement at the end of the introduction that summarized its main points, which helped transition to the “roadmap” that outlined the two studies we conducted. We considered incorporating the two references you mentioned, but upon reading them, failed to grasp their relevance to the current research.

*Study 1*

*1. It's important to clarify whether the participant pool was limited to undergraduate students or if it included individuals from other backgrounds.*

**RESPONSE:** In the methods sections of both studies, we specify that “Participants were XXX undergraduate students…” To remove any doubt of the subject population we have clarified that the participant pool in each study “was limited to undergraduate students.”

*2. Specify if there were any inclusion criteria other than being an undergraduate student (e.g., age range, academic major) to provide a clearer picture of the sample.*

**RESPONSE:** We have clarified in the methods section to each study of the revised manuscript that “No other inclusion criteria were used.”

*3. Ensure clarity in the description of the manipulation process. For instance, it's mentioned that participants were provided with false feedback about their status and others', but it's not entirely clear how this feedback was presented and if there were any variations in presentation among conditions.*

**RESPONSE:** To clarify the status manipulation process, we have added two appendices in the revised manuscript with the exact details of the process and have clarified what information was presented to all participants and what information was provided just to those in specific conditions.

*4. Clarify the rationale behind using a 1 to 7 scale for status ratings and ensure that this choice aligns well with the manipulation and the constructs under investigation.*

**RESPONSE:** In the methods section of Study 1, we have further clarified that “We used a 1 to 7 scale because that is the most used scale in status research *in psychology (e.g., Anderson et al., 2001; Cheng et al., 2013).*” The definition of status used in the manipulations “*That is, how much should each member be respected and admired in the group, how much should they lead the group’s task, and how much should others voluntarily defer to them?*” (see Appendices), is consistent with the definition of status used in the introduction (second sentence): “*Status is the respect, admiration, and voluntary deference individuals are afforded by others*.”

*5. Provide details on the selection and construction of attention check questions to ensure they effectively measure participants' engagement and attentiveness.*

**RESPONSE:** In the revised manuscript, we have clarified what attention checks were used to ensure participants were attentive: “During the initial questionnaire, described below, two attention check questions were included that asked participants to select specific responses, i.e., “If I am paying attention to this study, I will select the agree a little option,” and “If I am paying attention I will select this answer. /I will not select this answer. This method of detecting inattentive participants has been used extensively in past research to increase the quality of data (e.g., Shamon & Berning, 2020).” We note that attention checks of this nature are very common in psychological research (e.g., Huan & Bowling, 2015; Kam & Meyer, 2015; Meade & Craig, 2012; Shamon & Berning, 2020).

Huang JL, Bowling NA, Liu M, Li Y. Detecting insufficient effort responding with an infrequency scale: Evaluating validity and participant reactions. Journal of Business and Psychology. 2015;30:299-311.

Kam CCS, Meyer JP How careless responding and acquiescence response bias can influence construct dimensionality: The case of job satisfaction. Organizational research methods. 2015;18(3):512-541.

Meade AW, Craig SB. Identifying careless responses in survey data. Psychological methods. 2012;17(3):437.

Shamon H, Berning CC. Attention Check Items and Instructions in Online Surveys with Incentivized and Non-Incentivized Samples: Boon or Bane for Data Quality?. Survey Research Methods*.* 2020*;*14(1):55-77.

*6. Provide effect sizes (e.g., Cohen's d) for all significant findings to supplement the significance testing and enhance the interpretation of results.*

**RESPONSE:** Thank you for highlighting. Effect sizes (Cohen’s d and partial eta squared) have been added to all significant results in both studies in the revised manuscript.

*7.* *Consider discussing any potential limitations of the study, such as the generalizability of findings beyond the undergraduate student population or the potential impact of demand characteristics on participants' responses.*

**RESPONSE:** We have added a limitation section to the General Discussion rather than to each study which highlights these potential issues. Specifically, we comment “*A potential limitation of the current studies is the reliance on undergraduate student populations and the potential generalizability of the findings to other subject populations, although we note that prior research on status and well-being suggests findings in this domain do appear to generalize (e.g., Anderson et al., 2012). Another limitation is the potential for demand effects where participants may have guessed the hypotheses and be prompted to behave in a certain manner. The debriefing in both studies did not uncover this issue but future research should consider alternative non-computer mediated designs to remove this possibility.*”

*8. Provide detailed descriptions of the figures and tables in the text to guide readers in understanding the key findings presented in visual format.*

**RESPONSE:** Legends have been added to the Figures describing the conditions and means to help the reader understand the methodology used and what the results describe.

*9. Ensure that the study adheres to ethical guidelines, particularly regarding informed consent, debriefing procedures, and handling of participant data.*

**RESPONSE:** As per our response to the AE, we have clarified in the Methods section of Study 1 that “We have included the full ethics statement in the Methods sections of Study 1. Specifically, we have edited the prior statement to now read: “*The research in both studies was conducted in accordance with the principles expressed in the Declaration of Helsinki and the protocol was approved by The Committee for Protection of Human Subjects comprising the Institutional Review Boards of the University of California, Berkeley (IRB Approval ID: 2015-01-7044). All subjects gave their informed consent for inclusion before they participated in the study. and were then debriefed at the end of the study*.”

*Study 2*

*• Provide more detailed information about the rationale behind the sample size selection and power analysis. Justify why 400 participants were targeted for the study.*

**RESPONSE:** More detailed information has been added to the Methods section of Study 2, justifying the sample size used. Specifically, we now state “*A power analysis based on the effect sizes observed in Study 1 suggested that at least 72 participants were needed to achieve 80% statistical power (two-tailed; α = .05) for a two condition design (288 for an eight condition design). Given sample size recommendations for replication studies (Simmons et al., 2012), we increased the target sample size to 400 participants, consistent with the target sample size of roughly 50 participants per condition used in Study 1.”*

*• Consider providing more information on the demographic characteristics of the participants beyond just race and gender. Factors such as socioeconomic status, cultural background, and education level may influence subjective well-being and could be relevant to the study.*

**RESPONSE:** An interesting idea. Unfortunately, the only demographic characteristics we reported were those described in the manuscript including age, gender and ethnicity which were collected because they are usually reported in psychological studies. We didn’t collect additional demographic characteristics because they were not the focus of the current research.

*• it would be beneficial to include a rationale for choosing the specific values included in the values affirmation manipulation. Justify why these values were selected and how they relate to the study's objectives.*

**RESPONSE:** The values chosen were exactly those that Cohen and colleagues (2006) specified in their original self-affirmation manipulation as detailed in the supplemental materials to their article in Science (link: <http://www-inst.eecs.berkeley.edu/~cs375/fa13/cs375/Science-2006-Cohen-1307-10.pdf>). We intentionally used this standard self-affirmation manipulation (their article is cited more than 1700 times) that has been extensively used in the research literature. We have clarified in the Methods section of Study 2 in the revised manuscript that the 12 values are those “… *that Cohen and colleagues specified.*”

Justification for the use of the self-affirmation intervention was included in the introduction, but to remind readers of why this intervention was chosen, we have now also included a comment at the Start of Study 2 methods. Specifically, we added “*To address this question, we examined whether allowing participants to self-affirm would help mitigate the effects of status on SWB as prior research suggests this intervention prompts individuals to be less interested in boosting their own status (Sivanathan & Pettit, 2010).*” Thank you for this suggestion.

*• Provide more details on the randomization procedure to ensure that participants were evenly distributed across conditions.*

**RESPONSE:** In the methods sections to both studies, we have added the following clarification: *“Participants were randomly assigned to condition using a randomizer in the computer-mediated survey flow which randomly assigned participants to one of four conditions evenly.”*

*• Ensure clarity in the presentation of results. Some sentences are convoluted and could be simplified for better comprehension.*

**RESPONSE:** We have further edited the Study to increase the comprehensibility of the results. If there are specific results that remain unclear please let us know.

*• When reporting statistical results, include effect sizes along with p-values to provide a more comprehensive understanding of the findings.*

**RESPONSE:** Again, thank you for highlighting. Effect sizes (Cohen’s d and partial eta squared) have been added to all significant results in both studies in the revised manuscript. P-values have also been added to all findings.

*• Consider including a table summarizing the main results to aid readers in quickly understanding the key findings.*

**RESPONSE:** A table of means (Table 1) has been added summarizing which mean SWB scores differ significantly between conditions within Study.

*• Provide more detailed interpretations of the results, particularly regarding the implications of the interaction effects observed in the study.*

**RESPONSE:** We provide a much more detailed interpretation of the interaction effect found in Study 2 between own and others’ status, to help clarify its meaning and its connection to relative deprivation research.

*• Propose avenues for future research based on the study's findings, including potential follow-up experiments or longitudinal studies to further explore the effectiveness of interventions on subjective well-being.*

**RESPONSE:** We elaborate in the discussion on directions for future research, including the possibility of exploring other possible interventions that might mitigate the impact of status on SWB.

*discussion*

*1. there are some instances where the phrasing could be clarified for better understanding. For example, in the sentence "That is, while the correlations observed between status and SWB in prior research might have stemmed from multiple causal forces," it could be clearer if you specify what these multiple causal forces might be.*

**RESPONSE:** We have provided examples of possible causal forces to clarify this argument. For example, happier people might attain higher status, or extraverted people might both be happier and attain higher status.

*2. When discussing Study 2, where the authors explored the potential moderating effect of self-affirmation on the relationship between status and SWB, it would be beneficial to clearly delineate the main findings. Make it explicit which aspects of the relationship were affected by self-affirmation and which were not.*

**RESPONSE:** We now make more explicit that the effects of participants’ own status on their SWB were not moderated by self-affirmation, and that the effects of others’ status on participants’ SWB were also not moderated by self-affirmation, before turning to the one finding that was moderated by the self-affirmation manipulation – namely, in the condition where participants’ and others’ status were higher. In that condition, participants experienced higher SWB when they self-affirmed than when they did not.

*3. it could be beneficial to delve deeper into potential alternative explanations. For instance, apart from the proposed causal effect of status on SWB, are there other variables or mechanisms that could explain the observed relationship? Discussing potential alternative explanations would strengthen the robustness of the findings.*

**RESPONSE:** We agree and elaborate on possible alternative explanations for the correlation between status and SWB at the beginning of the discussion section – namely, that there might be a causal effect of SWB on status, and that third variables (e.g., extraversion) might drive the correlation.

*4. Provide more detailed suggestions for future studies. For example, in addition to examining individual differences in the relationship between status and SWB, what specific aspects of personality or social context might moderate this relationship? Providing concrete research questions and hypotheses for future studies would enrich the discussion.*

**RESPONSE:** We have elaborated in the discussion on suggestions for future studies, such as testing other possible interventions that might mitigate the impact of status on SWB, particularly the impact of others’ status (e.g., perspective-taking interventions), and testing whether individuals differ in the degree to which they derive subjective well-being from their status, and whether this difference moderates the impact of status on subjective well-being.

*5. Discuss how cultural differences might influence the relationship between status and SWB. Are there cultural norms or values that could affect how individuals perceive and respond to status? Considering cultural variations would provide a more comprehensive understanding of the findings and their generalizability.*

**RESPONSE:** We wouldn’t expect the results to change significantly across culture given a recent and comprehensive review of the psychological effects of status across multiple literatures revealed the effects of status and specifically the desire for status appeared to be universal and applied across cultures (see Anderson et al., 2015). However, we have acknowledged the possibility of cultural variation in the general discussion of the revised manuscript. Specifically, we comment “*Relatedly, the current research was conducted in a Western context, and it is possible that the observed effects might be stronger or weaker in different cultural contexts. A recent and comprehensive review of the research literatures on status (Anderson et al., 2015) revealed that the psychology of status and specifically the desire for status appears to be universal, applying cross-culturally, but leaves open the possibility that the effects of status might be stronger in different contexts. Future research should examine this possibility.*”

Anderson C, Hildreth JAD, Howland L. Is the desire for status a fundamental human motive? A review of the empirical literature. Psychological bulletin. 2015;141(3):574.

*6. Conclude the discussion by summarizing the key findings, implications, and avenues for future research. Reinforce the significance of the findings in advancing understanding of the relationship between status and SWB, while also acknowledging limitations and areas for further exploration.*

**RESPONSE:** We now include a summary paragraph at the end of the discussion, which outlines the main findings, some of the most important implications of these findings, as well as the limitations of the current research.

**Reviewer 2 comments**

*Reviewer #2: The discussion section of the article feels somewhat abrupt and lacks depth in its analysis. The discussion transitions quickly between different ideas without providing a thorough exploration of each point. For instance, while the competitive orientation towards status is briefly mentioned, further elaboration on its theoretical implications and practical relevance is warranted. Additionally, the finding that self-affirmation does not significantly moderate the effect of status on SWB requires more nuanced interpretation and consideration of alternative explanations.*

**RESPONSE:** We have elaborated on the theoretical implications of the idea that the desire for status is inherently competitive. For example, it suggests one possible explanation for why status stratification pervades groups: people do not seek egalitarianism, but instead, seek to be higher in status than others (thereby seeking a stratification in status wherein they have higher status than others). It also suggests that a fundamental human desire is competitive in nature, which would make it the only one we know of (other fundamental motives, such as the need for belongingness, are not thought to be competitive). We also elaborated on the overall inability of self-affirmation to moderate the effects of status on subjective well-being, including the possibility that no interventions moderate those effects, and that other interventions should be examined.

*Also, I question the suitability of undergraduate students as a sample population for examining the hypothesis addressed in this study. Typically in their twenties and yet to embark on their professional careers, undergraduate students may not undergo significant status changes in the short term, potentially limiting the generalizability of findings. Moreover, it appears that the study overlooks addressing the limitation of generalizing conclusions drawn from the experiments.*

**RESPONSE:** We have added a comment in the General Discussion about the limitation of using undergraduate student pools. Specifically, we note “*A potential limitation of the current studies is the reliance on undergraduate student populations and the potential generalizability of the findings to other subject populations, although we note that prior research on status and well-being suggests findings in this domain do appear to generalize (e.g., Anderson et al., 2012)*. However, we note that the social psychological conception of status used in the current research is context specific and one’s status can change significantly in all of the groups to which one belongs and over relatively short periods of time. Many undergraduate students join multiple groups during their undergraduate years (e.g. fraternities, sports teams, professional clubs, non-profit /charity groups, etc.) and the amount of respect, admiration and voluntary deference individuals enjoy in these groups can vary radically during their tenure in these groups.

*6. PLOS authors have the option to publish the peer review history of their article (*[*what does this mean?*](https://nam12.safelinks.protection.outlook.com/?url=https%3A%2F%2Fjournals.plos.org%2Fplosone%2Fs%2Feditorial-and-peer-review-process%23loc-peer-review-history&data=05%7C02%7Cjdh362%40cornell.edu%7C16a98a46a4b54c331ba108dc69e97692%7C5d7e43661b9b45cf8e79b14b27df46e1%7C0%7C0%7C638501699849705625%7CUnknown%7CTWFpbGZsb3d8eyJWIjoiMC4wLjAwMDAiLCJQIjoiV2luMzIiLCJBTiI6Ik1haWwiLCJXVCI6Mn0%3D%7C0%7C%7C%7C&sdata=fCrZzvzzrZqH1D%2FtIHOUcoOPcAMeyWA4Wb6ysTaBhYw%3D&reserved=0)*). If published, this will include your full peer review and any attached files.*

*If you choose “no”, your identity will remain anonymous but your review may still be made public.*

***Do you want your identity to be public for this peer review?*** *For information about this choice, including consent withdrawal, please see our* [*Privacy Policy*](https://nam12.safelinks.protection.outlook.com/?url=https%3A%2F%2Fwww.plos.org%2Fprivacy-policy&data=05%7C02%7Cjdh362%40cornell.edu%7C16a98a46a4b54c331ba108dc69e97692%7C5d7e43661b9b45cf8e79b14b27df46e1%7C0%7C0%7C638501699849715202%7CUnknown%7CTWFpbGZsb3d8eyJWIjoiMC4wLjAwMDAiLCJQIjoiV2luMzIiLCJBTiI6Ik1haWwiLCJXVCI6Mn0%3D%7C0%7C%7C%7C&sdata=tyTsc8ZBjVZnfQXYpVYkmVzrKpn0O50zPv3fF7rS%2FL8%3D&reserved=0)*.*

*Reviewer #1: No*

*Reviewer #2: No*

*[NOTE: If reviewer comments were submitted as an attachment file, they will be attached to this email and accessible via the submission site. Please log into your account, locate the manuscript record, and check for the action link "View Attachments". If this link does not appear, there are no attachment files.]*

*While revising your submission, please upload your figure files to the Preflight Analysis and Conversion Engine (PACE) digital diagnostic tool,* [*https://pacev2.apexcovantage.com/*](https://nam12.safelinks.protection.outlook.com/?url=https%3A%2F%2Fpacev2.apexcovantage.com%2F&data=05%7C02%7Cjdh362%40cornell.edu%7C16a98a46a4b54c331ba108dc69e97692%7C5d7e43661b9b45cf8e79b14b27df46e1%7C0%7C0%7C638501699849723213%7CUnknown%7CTWFpbGZsb3d8eyJWIjoiMC4wLjAwMDAiLCJQIjoiV2luMzIiLCJBTiI6Ik1haWwiLCJXVCI6Mn0%3D%7C0%7C%7C%7C&sdata=lCbRwdX2vg6W5kxjDdluQDj4Nr%2Bnf7oLfn1NzNchiTM%3D&reserved=0)*. PACE helps ensure that figures meet PLOS requirements. To use PACE, you must first register as a user. Registration is free. Then, login and navigate to the UPLOAD tab, where you will find detailed instructions on how to use the tool. If you encounter any issues or have any questions when using PACE, please email PLOS at* [*figures@plos.org*](mailto:figures@plos.org)*. Please note that Supporting Information files do not need this step.*
